# Emerging Role of Nuclear Receptors for the Treatment of NAFLD and NASH

**DOI:** 10.3390/metabo12030238

**Published:** 2022-03-11

**Authors:** Ryan D. Welch, Cyrielle Billon, McKenna Losby, Gonzalo Bedia-Diaz, Yuanying Fang, Amer Avdagic, Bahaa Elgendy, Thomas P. Burris, Kristine Griffett

**Affiliations:** 1 Biology and Chemistry Department, Blackburn College, Carlinville, IL 62626, USA; ryan.welch@blackburn.edu; 2 Center for Clinical Pharmacology, University of Health Sciences and Pharmacy and Washington University in St. Louis, St. Louis, MO 63110, USA; cyrielle.billon@uhsp.edu (C.B.); gonzabedia@gmail.com (G.B.-D.); fangyuanying@163.com (Y.F.); amer.avdagic@gmail.com (A.A.); bahaa.elgendy@uhsp.edu (B.E.); 3 Biochemistry, Biophysics and Structural Biology, School of Medicine, Washington University in St. Louis, St. Louis, MO 63110, USA; mckenna.losby@wustl.edu; 4 Department of Anesthesiology, School of Medicine, Washington University in St. Louis, St. Louis, MO 63110, USA; 5 UF Genetics Institute, University of Florida, Gainesville, FL 32611, USA; burris.thomas@ufl.edu; 6 Department of Anatomy, Physiology and Pharmacology, College of Veterinary Medicine, Auburn University, Auburn, AL 36849, USA

**Keywords:** non-alcoholic fatty liver disease, non-alcoholic steatohepatitis, nuclear receptors, lipogenesis, metabolism, inflammation, glucose metabolism, oxidative stress, insulin sensitivity, fibrosis, therapeutics

## Abstract

Non-alcoholic fatty liver (NAFLD) over the past years has become a metabolic pandemic linked to a collection of metabolic diseases. The nuclear receptors ERRs, REV-ERBs, RORs, FXR, PPARs, and LXR are master regulators of metabolism and liver physiology. The characterization of these nuclear receptors and their biology has promoted the development of synthetic ligands. The possibility of targeting these receptors to treat NAFLD is promising, as several compounds including Cilofexor, thiazolidinediones, and Saroglitazar are currently undergoing clinical trials. This review focuses on the latest development of the pharmacology of these metabolic nuclear receptors and how they may be utilized to treat NAFLD and subsequent comorbidities.

## 1. Introduction

**Figure 1:**
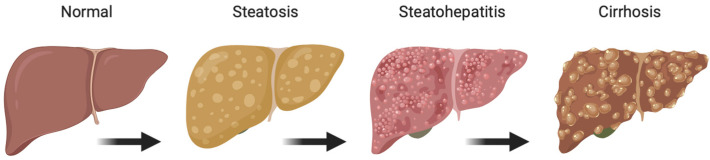
Stages of non-alcoholic fatty liver disease (NAFLD). Figure created with BioRender (accessed on 7 February 2022).

Non-alcoholic fatty liver (NAFLD) is the most prevalent chronic liver disease worldwide. NAFLD comprises a spectrum of diseases from simple steatosis (greater than 5% hepatic fat) to steatohepatitis (combination of lipid accumulation and inflammation and/or fibrosis) (Figure 1) [1,2,3,4,5]. In some cases, this disease can progress into advanced-stage liver diseases including cirrhosis or hepatocellular carcinoma (HCC). NAFLD is often associated with other metabolic disorders including obesity, type II diabetes, and cardiovascular diseases (including atherosclerosis). Considering the continued increase in patient BMI, it is no surprise that NAFLD has become so prevalent. In fact, obesity appears to play a significant role in both the development and progression of NAFLD and increases the chance that patients will develop non-alcoholic steatohepatitis (NASH) and fibrosis [6,7,8,9,10]. While approximately 15–20% of NAFLD patients are not considered obese, all NAFLD patients clearly demonstrate dysregulation of metabolic processes, lipid storage, endothelial damage, and increased expression of inflammatory markers [1,2,6,11,12,13,14,15].

**Figure 2:**
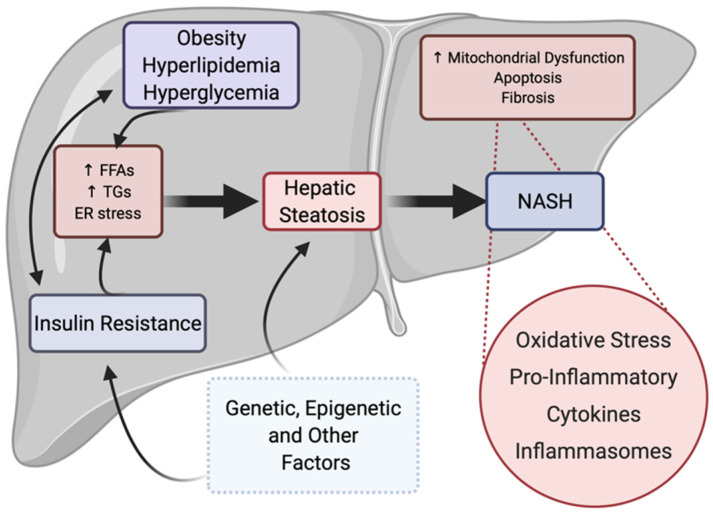
“Multi-hit” process of NAFLD progression. Insulin resistance, obesity, hyperlipidemia, and other factors may act independently or collaboratively to initiate the pathogenesis of fatty liver disease, characterized by increased hepatic triglyceride storage and steatosis. Disease progression may also be multifactorial, but most often occurs upon increased proinflammatory cytokine activation and increased hepatic oxidative stress, leading to NASH with fibrosis. Genetic, epigenetic, environmental, and other factors also play a role in the development of primary factors as well as in the development of steatosis. Figure created with BioRender (accessed on 7 February 2022).

NAFLD is a complex multifactorial disease associated with genetic, epigenetic, and environmental factors, with a pathogenesis that not only differs from patient to patient but remains unclear. Often, a “multi-hit” model is used to describe the development and progression of NAFLD, where the accumulation of hepatic triglycerides (steatosis) via increased lipogenesis and impaired free fatty acid degradation, in many cases as a result of insulin resistance and obesity, leads to the pathogenesis of fatty liver disease [16,17,18,19]. The accumulation of hepatic lipids leads to the activation of proinflammatory molecules and oxidative stress, which itself can lead to increased mitochondrial dysfunction, fibrosis, and NASH (Figure 2).

Oxidative stress is the imbalance between reactive oxygen species (ROS) production and the scavenging capacity of the antioxidant system. ROS include hydrogen peroxide and superoxide free radicals that are produced as byproducts of energetic metabolism in different types of liver cells. Steatosis induces the overproduction of ROS, which causes oxidative modifications to DNA, lipids, and proteins. These damaged macromolecules can accumulate and induce liver injury, cell death, inflammation, and promote metabolic dysfunction [20,21,22,23,24]. Targeting oxidative stress in addition to other factors (steatosis, inflammation, etc.) may be a viable therapeutic option for NAFLD.

The innate immune response is an important component of the immune system that recognizes and responds to potential pathogens in a “generic” fashion. In contrast to the adaptive immune system, the innate immune system does not confer long-lasting/protective immunity. It is typically considered the first line defense and displays the ability to discriminate against host vs. non-host/pathogen. Specifically, inflammasomes, that are quite distinct in that they form a high molecular weight caspase-1 activating complex, control the maturation and secretion of Interleukin 1β (IL-1β) and Interleukin 18 (IL-18); these cytokines play an essential role in the inflammatory response involved in the development and pathogenesis of fatty liver diseases (Figure 2) [25,26,27,28,29,30,31,32]. Unlike most cytokines that are translated into their active forms, both IL-1β and IL-18 are produced in a non-active precursor format and must be activated by proteolysis before they can be secreted in their active forms. Inflammasomes are “gatekeepers” of IL-1β and IL-18 activity since caspase-1 proteolytic activity associated with the multiprotein inflammasome regulates the processing and activation of these two cytokines. Given the significant efficacy of IL-1β and IL-18 in producing inflammation, their availability is controlled at two levels. Synthesis of pro-IL-1β and pro-IL-18 mRNA is tightly regulated at the transcriptional level via proinflammatory signaling mediated by nuclear factor kappa B subunit 1 (NF-κB) and/or mitogen-activated protein (MAP) kinase signaling pathways. Low levels of these precursor cytokines are maintained in cells providing minimal substrate if the inflammasome alone is activated. Similarly, activation of transcription of these two pro-cytokines cannot produce active cytokines without the activation of the inflammasome. Three NOD-like receptors (NLR) containing inflammasomes have been demonstrated to have physiological roles and they are named for the NLR protein they are associated with: NLRP1, NLRP3, and NLRC4. Each of these inflammasomes is stimulated by danger-associated molecular patterns (DAMPs) and pathogen-associated molecular patterns (PAMPs) with the specific regulatory mechanisms by assembling an oligomerized multiprotein complex that includes pro-caspase-1 and allows for the autocleavage of pro-caspase-1 to activate caspase-1 and process the pro-IL-1β and pro-IL-18 [26,32,33].

Inflammasomes are often activated in liver diseases by a variety of signals including cholesterol crystals, ROS, and palmitic acid. The role of inflammasomes in liver disease has been attributed to their expression in Kupffer cells and their capacity to initiate inflammation via the proinflammatory cytokine IL-1β. IL-1β promotes the recruitment of inflammatory cells to the liver, in addition to activating hepatic stellate cells (HSCs), which initiates fibrosis [25]. In the context of liver disease, the NLRP3 inflammasome has been implicated in many studies as the primary activator of inflammation and initiates the development of NASH in both rodent models and humans [26,31,32,33]. The activation of NLRP3 is mediated through liver parenchymal cells as well as hepatic immune cells. Recent mouse studies have shown that in animals with early stages of NAFLD, steatosis is established, however, without inflammation. Interestingly, these animals had no signs of NLRP3 inflammasome activation in the liver at the time of this study. The idea that liver-specific NLRP3 activation is required and essential for the progression of liver disease towards NASH and beyond is very interesting. Additionally, it is only when the NLRP3 inflammasome becomes activated in additional tissues and organs that metabolic syndrome is observed in mammals. Several nuclear receptors can regulate the expression of the NLRP3 inflammasome as well as other proinflammatory cytokines, thus providing promising targets for therapeutic evaluation.

Nuclear receptors (NRs) are a class of proteins that regulate the expression of genes responsible for a variety of different processes such as metabolism, homeostasis, inflammation, development, and many others. NRs are grouped into subfamilies (NR1, NR2, NR3, NR4, NR5, NR6, and NR0) based on their DNA-binding characteristics. In humans, there are forty-eight NR family members that act as ligand-activated transcription factors that respond to a variety of signals from steroid hormones, vitamins, and sterol metabolites [34,35]. Approximately half of these receptors are classified as orphan receptors, as they do not have well-characterized endogenous ligands. However, NRs with characterized ligands are targeted for the development of synthetic therapeutics to treat a myriad of diseases including diabetes, reproductive disorders, inflammation, and metabolic diseases.

The basic structure of a nuclear receptor consists of domains each having a unique role: N-terminal domain, transcription regulation domain, DNA binding domain (DBD), hinge domain, ligand binding domain (LBD), and C-terminal domain. The DBD is the most conserved domain that contains a rich number of cysteines and basic amino acids. The position of the cysteines is conserved among receptors and their arrangement of Zn^2+^ ions within the two zinc-finger structures located within the domain [34]. The binding of a nonpolar molecule to the ligand binding domain induces conformational changes that seem to control these properties and influence gene expression. The conformational changes that accompany the transition between the ligand-bound and ligand-unbound forms of the nuclear hormone receptors significantly affect their affinity for other proteins. The hinge domain is located between the DNA binding domain and ligand binding domain and is a short region with low conservation that functions in the modulation of DNA binding.

NRs usually function as ligand-dependent transcription factors but there are several of them, known as orphan nuclear receptors, that do not have any endogenous or synthetic ligands yet discovered. Ligands are nonpolar molecules that diffuse through cell membranes to bind to nuclear receptors at their ligand binding domain. NRs recognize specific DNA-response elements in the promoters and enhancers of their target genes and respond to ligands by altering their recruitment of different proteins such as coactivators and corepressors which will then change the expression of the regulated genes. Most NRs will function as dimers but there is a subset that function as monomers, and this includes many of the orphan members. Even without the binding of their ligands, NRs will bind their response elements and have an active role in regulation of their genes, and this is known as the basal level of expression. This basal level of expression can be regulated through the introduction of various synthetic ligands such as agonists, antagonists, inverse agonists, and partial agonists. Presence or absence of specific ligands affects the NR’s ability to recruit different transcriptional proteins by altering the gene expression.

Due to the vast amount of knowledge regarding a ligand’s effect on NRs, there has been much focus on the development of small-molecule synthetic ligands that can either have the same effect as endogenous ligands or the opposite effect. Synthetic ligands that mimic endogenous ligands are known as agonists, and ones that have the opposite effect are known as antagonists. For most NRs, agonists will bind to the ligand-binding domain causing conformational changes that recruit coactivator proteins which cause an increase in transcription of the NR’s target genes, whereas an antagonist binds the ligand binding domain and will prevent conformational changes such as the changes that the agonist causes to prevent coactivator recruitment, therefore preventing upregulation of gene expression. Inverse agonists are also explored which result in a conformational change that will reduce the basal levels of activity. This inverse agonist induces a conformational change that recruits a corepressor which then leads to the silencing of target genes. The defining characteristics of a ligand are not as definitive as there are partial agonists which will bind to the ligand binding domain and cause a partial activation of target gene transcription.

Here, we describe the roles that several NRs play in the liver physiology, metabolism, inflammation, and potential therapeutic strategies, including those that are currently in clinical trials, for the treatment of NAFLD.

## 2. Nuclear Receptor Targets for NAFLD

### 2.1. FXR

The farnesoid X receptor (FXR) is a bile acid receptor that regulates triglyceride metabolism via modulation of hepatic lipogenesis. FXR is highly expressed in the liver, intestine, and kidney. Bile acids modulate lipid metabolism and can cross the plasma membrane through simple diffusion or facilitated transport where it then binds to the LBD of FXR. FXR heterodimerizes with RXR and binds to inverted repeats with 1 nucleotide separating (IR1) [36]. Natural ligands of FXR include chenodeoxycholic acid (CDCA) and cholic acid (CA) [36]. Binding to natural ligand facilitates coactivator recruitment and upregulation of transcription. Synthetic ligands have also been designed targeting FXR including GW4064, which is commonly used as a positive control in many studies [36,37]. Recent research has shown that FXR is a major modulator of insulin sensitivity and lipid metabolism in animal models [15].

**Figure 3:**
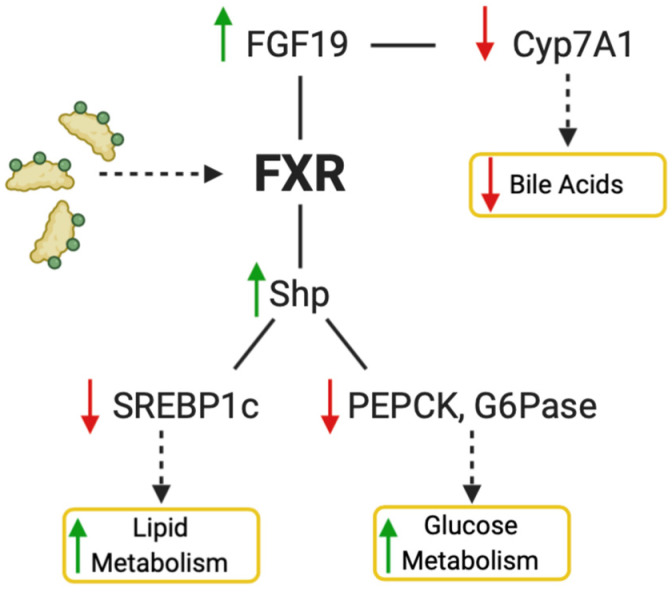
Activation of FXR by natural or synthetic ligands decreases bile acid production and increases lipid and glucose metabolism. For the bile acid pathway, FGF19 is upregulated and *CYP7A1* downregulated, which in turn causes a decrease of bile acid synthesis. Activation of FXR also increases SHP, which decreases *SREBP1c*, *PEPCK*, and *G6Pase*, causing an increase in lipid metabolism and glucose metabolism. Figure created with BioRender (accessed on 7 February 2022).

FXR is a master regulator of bile acid metabolism, lipid metabolism, and hepatic glucose metabolism. In patients with NAFLD, triglycerides accumulate in the liver leading to steatosis from increased de novo lipogenesis and fatty acid uptake in addition to reduced fatty acid oxidation and very-low-density lipoprotein (VLDL) export [36]. Activation of FXR in hepatocytes is protective against steatosis by decreasing lipogenesis and increasing fatty acid oxidation. FXR upregulates fibroblast growth factor 19 (FGF19) upon activation, which downstream leads to suppression of bile acid synthesis. The suppression of bile acid synthesis occurs by downregulation of cytochrome P450 family 7 subfamily A member 1 (*CYP7A1*) which is the rate-limiting step in bile acid synthesis from cholesterol (Figure 3). Lipogenesis is modulated through decreased expression of sterol regulatory element binding protein 1 (*SREBP1c*) upon activation of FXR which induces small heterodimer partner (SHP) [37]. Hepatic glucose metabolism is also modulated through FXR activation by reducing levels of peroxisome proliferator-activated receptor gamma coactivator 1 alpha (PGC1α), phosphoenolpyruvate carboxykinase (*PEPCK*), and glucose-6-phosphatase (*G6Pase*) [38]. PGC1α is activated by cAMP response element-binding protein (CREB), then acts as a coactivator for nuclear receptors glucocorticoid receptor (GR) and hepatic nuclear factor 4 (HNF4) that are involved in gluconeogenesis. FXR and SHP are known to bind to the promoter of the *PEPCK* gene to regulate glucose metabolism [39].

Humans with NASH been found to have elevated levels of bile acid production, which, in turn, can cause progression of inflammation and fibrosis by inducing oxidative stress. Rats fed a high fat diet have increased *CYP7A1* and bile acid production, showing the potential of modulating FXR as a therapeutic for this disease [40]. FXR knock out mice not only have increased bile acid production, but also a NASH phenotype of steatosis, inflammation, and fibrosis. Alternatively, activating FXR has been shown to be protective against inflammation through NF-kB pathway and monocyte chemoattractant protein-1 (MCP-1) [41]. It has also been shown that activation of FXR-SHP regulatory pathway helps inhibit the progression of fibrosis by inhibiting hepatic stellate cells [42].

**Figure 4:**
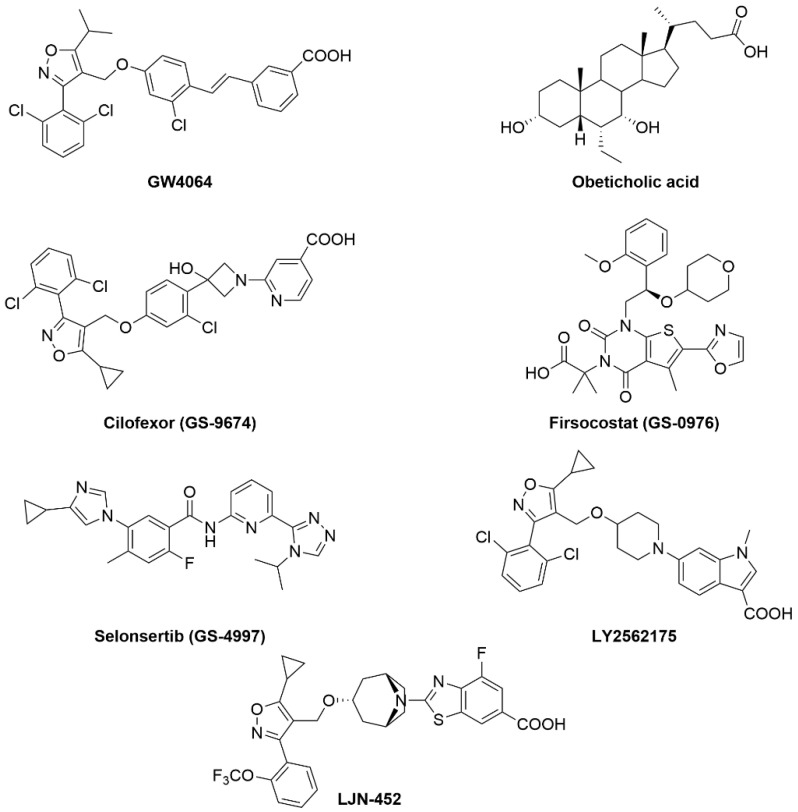
Chemical structures of FXR agonists that are currently in clinical trials.

As previously mentioned, GW4064 is a potent synthetic non-steroidal FXR agonist (Figure 4). Through many experiments by different groups, it has been shown that GW4064 reduces hepatic lipid accumulation, steatosis, and improves hyperglycemia and hyperinsulinemia [36]. However, this ligand has poor bioavailability, which halted its potential as a clinical candidate. Obeticholic acid is an FXR agonist that is currently being studied for treatment of NAFLD. In phase 2 clinical trials, obeticholic acid showed improvements in liver inflammation and fibrosis. Additionally, obeticholic acid decreased NAFLD activity score (NAS) scores by ≥2 points without worsening fibrosis in patients [43]. These trials observed worsening dyslipidemia—increased LDL—in some patients that can be managed by co-administrating statins but raises some concerns [6].

Cilofexor (GS-9674) is a potent FXR agonist (EC_50_ = 43 nM) (Figure 4) with a potential for the treatment of NASH due to its anti-inflammatory and anti-fibrotic effects. A phase 2 study to evaluate the safety and efficacy of Cilofexor (NCT02854605) was completed recently. The study showed that Cilofexor was well tolerated when dosed orally at 30 and 100 mg for 24 weeks. After 24 weeks, significant reduction in serum gamma-glutamyltransferase, serum bile acids, and hepatic steatosis was observed in patients with NASH. For NASH patients who received the 100 mg dose of Cilofexor, 39% showed ≥30% decline in magnetic resonance imaging-proton density fat fraction (MRI-PDFF), while only 14% of patients who received the 30 mg dose showed the same level of MRI-PDFF reduction [44]. Additionally, Cilofexor (GS-9674) is under evaluation in phase 2 clinical trials for treatment of NASH (NCT02781584) in combination with Firsocostat (GS-0976) and Selonsertib (GS-4997) [45,46,47,48].

Firsocostat (GS-0976) (Figure 4) is an acetyl-CoA carboxylase (*ACC*) inhibitor with potential to reduce hepatic steatosis and improve insulin sensitivity [49]. In preclinical studies, combination of Cilofexor and Firsocostat was more effective than using Cilofexor as monotherapy for the treatment of NASH. Twenty patients received both Cilofexor (30 mg) and Firsocostat (20 mg) orally for 12 weeks. They showed improvement in hepatic steatosis, liver stiffness, alanine aminotransferase, γ-glutamyltransferase, and serum markers of hepatic fibrosis. The combination therapy was safe and led to reduction in hepatic proton density fat fraction (PDFF) and hepatic de novo lipogenesis (DNL) [47,50].

Selonsertib (GS-4997) is a selective inhibitor of apoptosis signal-regulating kinase-1 (ASK1) with potential anti-fibrotic and anti-inflammatory activities [51]. This drug failed previously in phase 3 studies on NASH patients with fibrosis (stage 3) and cirrhosis (stage 4). Selonsertib is currently in phase 2 study in combination with Cilofexor and Firsocostat (NCT03449446). This study is underway on 395 patients with severe fibrosis or compensated cirrhosis due to NASH.

Tropifexor (LJN452) is potent non-bile acid FXR agonist that was advanced into phase 2 human clinical trials in patients with NASH [NCT02855164] and PBC [NCT02516605] [52]. In healthy volunteers, Tropifexor was found to be safe and well tolerated. TERN-101 (LY2562175) is another potent non-bile acid FXR agonist with remarkable lipid modulating properties. This drug effectively lowers both LDL and triglycerides while raising HDL [53]. Moreover, LY2562175 possess good PK properties and has advanced into phase 2 clinical trial for the treatment of NASH. EDP-305 is a second-generation non-steroid FXR agonist used for the treatment of NASH. ARGON-1 phase 2a study of EDP-305 (NCT03421431) was completed and showed significant reduction in ALT and liver fat content at 2.5 mg dose. The major side effects reported from this trial were pruritus, headaches, dizziness, and GI-related symptoms. The results of these clinical trials are summarized in Table 1.

### 2.2. PPARs

**Figure 5:**
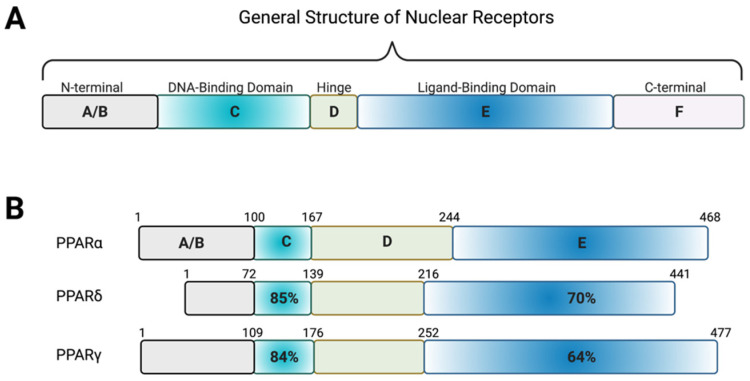
Homology of the PPAR receptors. (**A**) Schematic showing the general structure of nuclear receptors. The N-terminal A/B region contains the ligand-independent activation of function-1 region, which is highly variable among the nuclear receptors. The DNA-binding domain consists of two zinc fingers that can recognize and bind specific sequences of DNA or response elements. This region is highly conserved among the nuclear receptors. The highly variable hinge region connects the DNA-binding domain to the ligand-binding domain, a hydrophobic region consisting of alpha helices that bind natural or synthetic ligands to induce transcriptional regulation of target genes. In most nuclear receptors, the ligand-binding domain contains the ligand-dependent activation of function-2 region, which is important in the recruitment of co-activators. At the C-terminal, there is often a highly variable region present which aids in the stabilization and recruitment of co-activators. (**B**) The three PPAR isoforms share amino acid sequence homology of the DNA-binding and ligand-binding domains. Figure created with BioRender (accessed on 7 February 2022).

**Figure 6:**
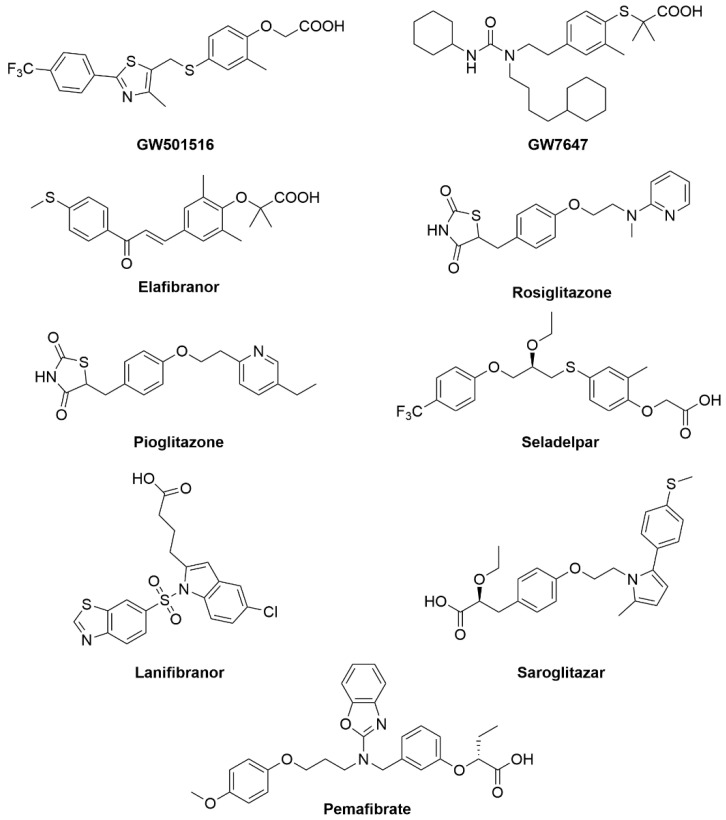
PPAR compounds that are currently in clinical trials or have approval for the treatment of NAFLD and related disorders.

Peroxisome proliferator-activated receptors (PPARs) exist in three isoforms (alpha, beta/delta, gamma), all of which require heterodimerization with the retinoid x receptor (RXR) to bind to DNA response element characterized by a direct repeat of an AGGTCA “half site” with 1 nucleotide separating the half sites (DR1) [54]. They share 60–70% amino acid sequence identity in their LBDs and have larger ligand-binding pockets than most NRs (Figure 5) [55,56]. PPARα is expressed in the liver and skeletal muscle while PPARδ is ubiquitously expressed [57,58]. PPARγ is expressed predominantly in adipocytes but is also found in other tissues, including the liver [59]. The natural ligands for this NRs are believed to be unsaturated fatty acids [60]. Upon binding of a natural ligand, transcriptional coactivators are recruited, and transcription activated. Many synthetic ligands have been designed targeting PPARs including GW501516 (specific agonist for PPARδ), GW7647 (specific agonists for PPARα), and thiazolidinediones (TZDs) that specifically target PPARγ (Figure 6).

PPARα being the most abundant isoform in the liver has been a logical target for the treatment of NAFLD. PPARα is involved in regulating lipid metabolism, inflammation through regulation of NF-kB, and gluconeogenesis and autophagy during starvation [61,62,63]. Several PPARα selective agonists have been evaluated in clinical trials including clofibrate, fenofibrate, and gemfibrozil, but have not been successful in improving histological markers of steatosis and inflammation [9].

Seladelpar (MBX-8025), is a PPARδ agonist that was evaluated in phase 2 clinical trials for NASH and PSC. Despite early promising results of this drug in reducing ALT and LDL, the trials were terminated due to observed liver damage in NASH patients [63]. Saroglitazar (Lipaglym) is a dual PPARα and PPARγ agonist that was approved for use in India for treating diabetic dyslipidemia in Type II diabetics. This drug is currently in multiple clinical trials in USA for the treatment of NAFLD (phase 2) and NASH (phase 3) [64,65,66]. Saroglitazar was approved in India on March of 2020 for the treatment of NASH, which make it the first approved drug for NASH in the world. In phase 3 trials in India, the drug reduced both liver fat and liver enzymes (Figure 6).

Elafibranor, a dual PPARα/δ agonist, had a successful phase 2b clinical trial showing improvements in defined endpoints such as decreased liver enzymes, plasma lipids, glucose homeostasis, and systemic inflammation (Figure 6) [62]. Unfortunately, in the Genfit phase 3 trial for NASH, it was found that elafibranor was not as efficacious as previously thought, showing only about a 19% response rate in patients as compared to placebo [63].

PPARδ is the least explored isoform of the three PPARs but has great therapeutic potential for treatment of NASH/metabolic disease due to its role in regulating lipids, glucose homeostasis, and fatty acid synthesis [9]. Many studies have been performed with the potent agonist GW501516 in vivo NASH models showing improved insulin sensitivity and steatosis. Although GW501516 is a potent agonist that shows improvements in NAFLD, further clinical development was terminated from cancer development in preclinical models [54]. Currently, PPARδ selective agonists are being explored by Mitobridge, Boston, MA, and are currently in clinical trials 1 and 2 for kidney disease, fatty acids oxidation disorders, and Duchenne’s muscular dystrophy.

There is a clear need for therapeutics for the treatment of NAFLD. Researchers have recently tapped into the NR superfamily to explore therapeutic capabilities for the treatment of NAFLD. For example, NAFLD is often diagnosed in addition to obesity and insulin resistance/Type 2 diabetes mellitus (T2DM). The prevalence of NAFLD in patients with T2DM is over 70%. A well-known target for the treatment of T2DM is PPARγ, by utilizing the thiazolidinediones (TZDs) family of therapeutics. TZDs typically act by decreasing insulin resistance via activating the PPARγ pathway. This upregulation of PPARγ leads to increased glucose uptake by peripheral tissues as well as lowered hepatic production of glucose. Several clinical studies in both diabetic and non-diabetic patients on TZD therapies suggested that TZDs hold utility in the treatment of NAFLD and NASH. The TZDs rosiglitazone and pioglitazone were tested in nine separate clinical trials of T2DM patients. The trials demonstrated that TZDs increase peripheral glucose clearance and improved insulin sensitivity while also appearing to significantly reduce hepatic fat accumulation and free fatty acid (FFA) concentration (Figure 6).

Lanifibranor is a pan-PPAR agonist and phase 2 clinical study (NCT03008070) to evaluate this drug for the treatment of NASH was completed recently. The drug reduced inflammation and did not worsen fibrosis in NASH patients. Phase 3 clinical studies of Lanifibranor is predicted to start in late 2020 or early 2021 [67]. Pemafibrate (K-877) is a potent and selective PPARα modulator that was approved in Japan for the treatment of hyperlipidemia [68]. This drug has passed phase 2 clinical trials (NCT03350165) to evaluate its efficacy and safety in patients with NAFLD and showed significant efficacy in the reduction of steatohepatitis that parallels animal data from earlier studies (Figure 6) [63]. The results of these clinical trials have been summarized in Table 1. With significant roles in lipid and glucose disorders, the PPAR receptors have proven to be a rich target for drug discovery. It is likely that some combination of PPAR therapeutic will be a standard treatment for NAFLD.

### 2.3. LXRs

**Figure 7:**
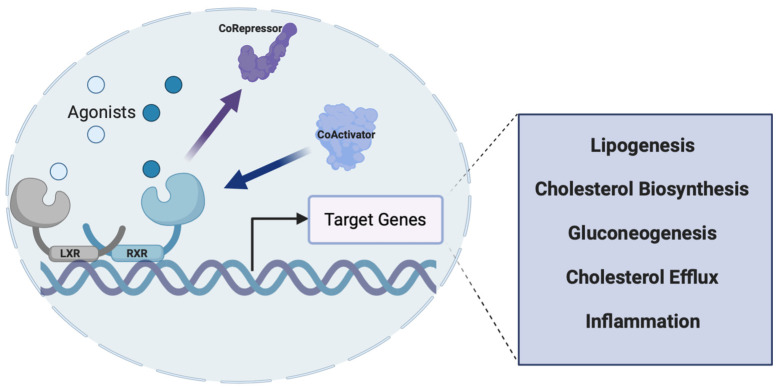
Mechanism of action of the LXR receptors. LXR heterodimerizes with RXR and recognizes specific DNA sequences (LXRE) within the promoter of its target genes. While there is some basal level of activity, upon agonist binding (natural or synthetic), a conformational change occurs that causes the recruitment of co-activators and allows for transcription of the target gene to occur. Genes involved in lipogenesis, cholesterol biosynthesis, gluconeogenesis, and inflammation are all regulated by LXRs, making this nuclear receptor a potential therapeutic target for NAFLD. Figure created with BioRender (accessed on 7 February 2022).

Liver X receptors (LXRs) are a group of ligand-activated transcription factors that occur in two isoforms LXRα (NR1H3) and LXRβ (NR1H2). Both isoforms were discovered during the 1990s and function as transcriptional regulators of cholesterol metabolism, de novo lipogenesis, and gluconeogenesis [69,70] and have similar homology to PPARs, FXR, and RXRs, and even share some functional activity. LXRα is highly expressed in liver, kidney, intestines, fat tissues, macrophages, lung, and spleen, whereas LXRβ is ubiquitously expressed. LXR regulates activity of target genes by forming obligate heterodimers with RXR and that LXR-RXR heterodimer binds to the LXR response element (LXRE) in the regulatory regions of the DNA. Upon binding of a ligand, a conformational change in the protein occurs and recruits co-activators which displaces the co-repressor that is usually bound to the dimers in the absence of an agonist/endogenous ligand, causing the recruitment of transcriptional machinery and downstream activation of target genes (Figure 7). LXR was an orphan nuclear receptor until in 1996, when Mangelsdorf and colleagues successfully de-orphanized the receptor. They discovered a specific group of endogenous oxysterols that were shown to activate transcription through LXRα. A GAL4-LXRα and GAL4-responsive luciferase reporter cotransfection system was used along with concentrated lipid extracts, that was prepared from a variety of tissues to identify potential LXRα ligands [69,71,72]. After conducting multiple sequence comparisons and phylogenetic analyses of the DBD (DNA-binding domain) and the LBD (ligand-binding domain), LXR was described as RLD-1 initially, a novel member of the thyroid/retinoid hormone receptor subfamily that heterodimerizes with RXR that recognizes a conserved direct repeat 4 (DR-4) response element, usually a variant of the idealized sequence AGGTCANNNNAGGTCA on the DNA [34,73,74,75]. Several co-transfection studies showed that this RLD-1/RXR/DR4 binding is constitutively active, and RLD-1 does not compete for RXR, which suggested that LXR (RLD-1) was different from RARs. Co-transfection studies revealed that transactivation of this LXR-RXR heterodimer was selectively induced by the addition of retinoic acids as well as by 9-cis-retinoic acid, which led Rainer and group to believe that LXR selectively activated the DR4 response element in the presence of RXR [74].

Oxysterols, which are oxygenated derivatives of cholesterol (22-(R)-hydroxycholesterol, 27-hydroxycholesterol and cholestenoic acid), have been identified as the endogenous ligands of LXR [69]. The oxysterols that activate LXRα are found endogenously at the rate-limiting steps of three major biological pathways: steroid hormone biosynthesis, bile acid synthesis, and the conversion of lanosterol to cholesterol [71,72,76,77]. This led to the discovery that LXR was indeed an important master regulator of sterol regulatory element binding protein 1 (*SREBP1*) and therefore played a major role in cholesterol sensing and fatty acid metabolism [78,79]. This was an important discovery since manipulation of this receptor could be used to treat a variety of cholesterol-related diseases, including atherosclerosis and other cardiovascular-related disorders. In 2003, the Burris group identified the first non-oxysterol natural product ligand of LXR, an indole alkoid fungal metabolite extracted from *Penicilium paxili*, paxiline [80]. Radioligand binding assays and scintillation proximity assays were employed to identify binding of paxiline to LXR. Paxiline was identified to be an efficacious natural ligand that was able to bind specifically to both LXRα and β. The alpha screens determined that the binding of paxiline led to the recruitment of the co-activator to the LXR-RXR heterodimer and activates LXR-mediated gene transcription of SREBP and ATP-binding cassette subfamily A member 1 (*ABCA1*) [80,81]. Unfortunately, paxiline is known to be a very potent antagonist to calcium-activated potassium channels. The drug showed toxicity in vivo due to its tremorgenic myotoxin property and was not further pursued.

LXR’s downstream target genes play a major role in lipid metabolism by regulating uptake, transport, absorption, and excretion of cholesterol and lipids in a tissue-specific manner. In the liver, LXR helps in the conversion of cholesterol into bile acids via *CYP7A1* [70]. LXR-mediated activation of target genes, such as *SREBP1c* [82], fatty acid synthase (*FAS*), carbohydrate response-element binding protein (*ChREBP*), acetyl CoA carboxylase (*ACC*) and stearoyl CoA desaturase 1 (*SCD1*), leads to increased lipogenesis [83,84]. Mice with disrupted LXRα expression displayed defective expression of *SREPB1c*, *ACC*, *SCD1*, and *FAS*, which demonstrated that LXR plays an important role in the lipogenic pathway. Based on the role LXR plays in cholesterol sensing and regulation of hepatic lipogenesis, it has been validated as a potential therapeutic target for fatty liver diseases.

**Figure 8:**
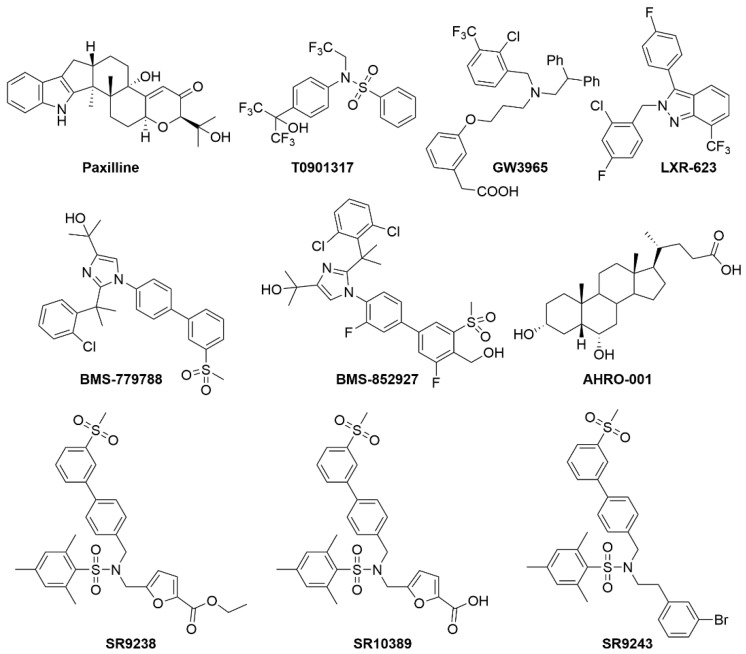
Known LXR modulators.

Over the past few years, it has been shown that LXR is not only a major player in lipid and cholesterol metabolism but is also involved in the inflammatory pathway. In macrophages, LXR plays a significant role in regulating reverse cholesterol transport via the *ABCA1* and ADP-ribosylation factor-like 7 (*ARL7*) gene which subsequently promotes the movement of cholesterol to the plasma membrane and efflux. Macrophages are known to play an important role in host defense and regulation of inflammatory responses; however, they also play an important metabolic role [85,86,87,88]. They are not only involved in phagocytic host defense against pathogens, but they are also involved in clearing apoptotic cells and oxidized lipoproteins from the system. Atherosclerotic plaques are caused by an inflammatory reaction to cholesterol-rich macrophages (foam cells) in the arteries. In these hypercholesterolemic conditions, the accumulation of cholesterol drives the conversion of macrophages into foam cells, causing the formation of atherosclerotic plaques [73,89]. To deal with these elevated levels of cholesterol, LXRs reduce cellular cholesterol by activating reverse cholesterol transport in peripheral cells by promoting cholesterol efflux via upregulation of ABC transporters [73,75]. Therefore, compounds that activate or stimulate the receptor have major potential in slowing down the progression of coronary heart disease. A benzenesulfoamide, T0901317 [*N*-(2,2,2-Trifluoroethyl)-*N*-[4-2,2,2-trifluoro-1-hydroxy-1-(trifluoromethyl)ethyl]phenyl]-benzenesulfonamide] (Figure 8), an LXR agonist, has been widely studied and characterized.

It was shown in 2002 that activating LXR had an atheroprotective effect on LDL receptor deficient mice (LDLR^−/−^). This synthetic LXR ligand was shown to significantly reduce atherosclerotic lesions in LDLR^−/−^ mice [90]. On teasing apart the entire mechanism, it was found that T0901317 increases the expression of *ABCA1* in these mice and subsequently increases reverse cholesterol transport. As discussed earlier, *ABCA1* regulates cholesterol absorption in the intestines. Patients with a mutation in their *ABCA1* gene have a deficiency in their high-density lipoprotein (HDL) and the condition is called Tangier disease. These patients have low HDL and severely high plasma cholesterol levels. They are at a much higher risk for atherosclerosis [14,91,92,93,94,95,96,97]. Therefore, pharmacologic activation of LXR with T0901317 showed decrease in atherosclerotic lesions, plasma cholesterol, and triglycerides in LDL receptor-deficient mice but on the other hand was also seen to induce high levels of hepatic lipogenesis, leading to hepatic steatosis in both LDLr receptor-deficient, diet-induced obese and diabetic (db/db) mice [91]. T0901317 has also been shown to act “promiscuously” with respect to nuclear receptor binding. In a Gal4 nuclear receptor profiling of T0901317, the compound showed promiscuity in binding with LXR, ROR, FXR, and PXR [98,99,100,101].

In addition to the T0901317 compound, a more specific synthetic LXR agonist GW3965 has been shown to improve glucose tolerance in rodents [102,103]. In rodent models of diet-induced obesity and insulin resistance, GW3965 (Figure 8) has been shown to regulate genes involved in glucose metabolism in the liver and the adipose tissue. GW3965 was revealed to inhibit gluconeogenesis in the liver and, in turn, increase the expression of glucokinase and the subsequent utilization of high glucose in the liver. In the adipose tissue, GW3965 mediates activation of LXR and leads to the increased expression of the glucose transporter, *GLUT4*, thereby increasing glucose uptake in the adipose tissue. GW3965 has also been shown to reciprocally regulate and reduce inflammation and increase lipid metabolism. GW3965 reduces inflammation by inhibiting inflammatory gene expression in mouse models of contact dermatitis and atherosclerosis [102,104].

The control of glucose metabolism is very closely tied with lipid metabolism. In white adipose tissue, LXR regulates the expression of apolipoprotein D (ApoD) and thyroid hormone-inducible hepatic protein (THRSP or also known as SPOT14), which are known to promote catabolism of fatty acids via β-oxidation pathway in the mitochondria [105,106,107]. LXR also plays a major role in inducing the expression of *GLUT4*, which is an important glucose transporter, thereby leading to increased glucose uptake in the adipose tissue. Even though these LXR agonists show potential pharmacologic properties, they also raise a couple of concerns. It is necessary to tease apart the promising effects of these LXR agonists on glucose metabolism from the effects on *SREBP-1c*. The interdependence of these two pathways would result in the suppression of gluconeogenesis, which would imply induction of de novo lipogenesis. These high levels of triglycerides would counteract the valuable effects of the glucose tolerance. Therefore, both T0901317 and GW3965 have, therefore, been shown to increase plasma and liver triglycerides and lead to profound hepatic steatosis, making these synthetic compounds unsuitable as a therapeutic agent.

Several LXR agonists were developed that went into clinical trials and ultimately failed. LXR-623 (Figure 8), currently owned by Pfizer pharmaceuticals, was the first published study of the effects of LXR ligands in humans [108]. LXR-623 entered phase 1 of the clinical trials for safety and majorly targeted at enhancing the reverse cholesterol transport, thereby playing a major atheroprotective role. They assessed the effects of their drug, administered orally on healthy participants. No deaths or severe adverse effects were reported from this study but 55% of the participants of this study experienced treatment-emergent adverse events (TEAE) that mostly included neurologic or psychiatric disorders such as lightheadedness, decreased comprehension, confusion, palpitation, and paresthesias. This project was terminated post-phase 1.

Compounds such as CS-8080 (company: Daichii Sankyo, Tokyo, Japan) and BMS-779788 and BMS-852927, also known as XL-652 and XL-041, respectively (company: Exelixis and Bristol-Myers Squibb, New York, NY, USA), were also designed to target LXR. These compounds have also been known to enter phase 1 clinical trials, but all the studies were terminated for undisclosed reasons. Hyodeoxycholic acid, currently AHRO-001 (company: AtheroNova, Los Angeles, CA, USA), is a bile acid derivative that targets LXR. This compound was shown to improve HDL function and have an atheroprotective effect on LDLR^−/−^ mice. Hyodeoxycholic acid (AHRO-001) is currently known to have successfully completed the phase 1 clinical trials.

In 2013, a novel synthetic compound, SR9238 (Figure 8), which acts as an LXR inverse agonist and is selective in the liver, was developed to target NAFLD [109]. SR9238 has been shown to display nanomolar efficacy, with IC50 of 214 nM for LXRα and 43 nM for LXRβ. The drug has shown significant selectivity to LXR and has not affected the expression of any other nuclear receptor. The compound has been shown to significantly suppress basal transcriptional activity of LXR and downregulate the expression of its target genes, especially fatty acid synthase (*FASn*) and *SREBP1c*, which are majorly involved in lipogenesis. An inverse agonist of LXR would suppress reverse cholesterol transport via the suppression of *ABCA1* expression. That would be detrimental and would place patients with fatty liver disease at an added risk of developing atherosclerosis. Therefore, synthesizing an inverse agonist that would be rapidly metabolized in the liver would provide extended liver exposure but no exposure to the peripheral tissues. The presence of an ester moiety on SR9238 contributes to its special ability of only targeting liver tissue selectively. Pharmacokinetic studies have revealed no signs of SR9238 in the plasma, brain, or skeletal muscle 2 h after administering 30 mg/kg of the drug intraperitoneally. SR9238 is metabolized into its acid analogue SR10389 by the plasma lipases. This acid analogue SR10389 has shown to have no effect on LXRα or LXRβ [109]. Liver-selective LXR inverse agonist SR9238 has been demonstrated to significantly reduce hepatic steatosis and development of NASH in obese, high-fat-diet-fed mice by suppressing hepatic lipogenesis, thus making it a promising candidate as a therapeutic agent for the treatment of NAFLD [110].

SR9243, another novel synthetic LXR inverse agonist, was developed based on the structure of SR9238. Unlike SR9238, SR9243 is not liver-selective. SR9243 was developed with the goal of targeting the Warburg effect and lipogenesis in cancer cells [111]. This drug was shown to significantly downregulate LXR-mediated glycolysis and lipogenesis selectively in a wide array of cancer cells. The compound also efficaciously induced cancer cell death without being toxic to non-malignant cells. It was also capable of sensitizing cancer cells to chemotherapeutic treatments. The drug was shown to have promising therapeutic effect in the field of developing cancer therapeutics. Further development of liver-specific LXR inverse agonists with enhanced pharmacokinetic and dynamic properties may prove to be a promising therapeutic agent for the treatment of NAFLD.

### 2.4. RORs

RAR-related orphan receptors (ROR) are members of the NR superfamily that are known to be involved in inflammatory and metabolic processes. The RORs represent a subfamily of NRs that includes three members: RORα, RORβ, and RORγ. RORs bind to the DNA as monomers to specific motifs known as ROR response element (RORE) and act as transcriptional regulators. RORα is widely expressed in immune cells, skeletal muscles, skin, lung, adipose tissue, brain, and liver [112,113,114,115]. RORβ mainly has expression in the brain, retina, and pineal gland [116]. RORγ is highly expressed in the thymus, muscle, testis, pancreas, prostate, heart, and liver [117]. RORs are constitutively active transcription factors, but oxygenated sterols may function as high affinity ligands. The 7-oxygenated sterols (7α-OHC, 7β-OHC, and 7-ketocholesterol) function as inverse agonists to both RORα and RORγ, modulating the expression of RORα/γ-dependent target genes [118]. Several other endogenous ligands have been described in the last 10 years [119]. Our lab and others have recently developed synthetic ROR ligands, both agonists and inverse agonists. The inverse agonist, SR3335, was initially identified based on its ability to inhibit the constitutive activity of RORα with little effect at RORγ and suppressed expression of RORα target genes involved in hepatic gluconeogenesis, including *G6Pase* and *PEPCK* [120]. SR1078, an RORα/γ agonist stimulated expression of two ROR target genes, *G6Pase* and fibroblast growth factor 21 (*FGF21*), in the liver. Pharmacokinetic studies revealed that SR1078 displays reasonable plasma exposure and can be used both in vitro and in vivo [121]. SR1001, a first-in-class RORα/γ-specific inverse agonist directly binds to the LBD of both RORα and RORγ, resulting in a conformational change that decreases affinity for coactivators and increases affinity for corepressors and has been shown to decrease Th17 cells differentiation both in vitro and in vivo [122]. Several thiourea derivatives, including JC1-40, have been identified as RORα agonists [123]. Huh et al. identified the well-known cardiac glycoside digoxin, as an inhibitor of RORγ activity [124].

The role of RORα in liver metabolism and NAFLD is very controversial and several studies and or mouse models have shown opposite results. The Stagger (RORα^sg/sg^) mice (6.5 kb genomic deletion of RORα gene) developed a severe ataxia but also have impaired glucose and lipid metabolism, inflammatory, and immune response [125,126]. RORα^sg/sg^ mice fed with high-fat diet are resistant to development of hepatic steatosis [125]. Whole-body RORα-deficient mice have improved metabolic profiling, decreased obesity under high-fat diet, and display an anti-inflammatory profile, with a decrease in plasma proinflammatory cytokines and lymphocytes CD4^+^ and CD8^+^ cell population in spleen [127]. Several groups have generated liver-specific RORα deletion with contradictory results. Kim and colleagues have shown that liver-specific RORα deletion induces NASH in mice under high-fat diet [128] but, on the other hand, Molinaro et al. report no increase in liver steatosis in a different model of liver-specific RORα deletion under a Western diet [129].

As NAFLD involve both hepatocytes and immune cells, liver-specific deletion of RORα may not be the best model to study its role in the disease. Myeloid-specific RORα-null mice are more susceptible to HFD-induced NASH due to decreased M2 polarization of Kupffer cells, decreasing interleukin 10 (IL-10) and increasing tumor necrosis factor alpha (TNFα) and interleukin 6 (IL-6) production, leading to lipid accumulation and hepatocytes apoptosis. Moreover, treatment with RORα agonists promoted M2 polarization and improved NASH symptoms in mice [130]. HFD-fed mice treated with RORα agonist JC1 showed attenuation of hepatic steatosis due to activation of AMPK signaling in the liver [131]. Several human datasets (GSE33814 and GSE89632) show a reduction of RORα expression in NAFLD patients [132,133]. Proinflammatory genes (*Arginase I* and *CD36*) are RORα target genes, and their expression are reduced in NAFLD patients [134]. On the other hand, some of the RORα target genes involved in fatty acid oxidation, such as *FASn* and *Srebf1*, are upregulated in NAFLD patients. As RORα regulates a variety of metabolic and inflammatory components, continued research is needed to understand the role of this receptor in NAFLD and whether it may be a valid therapeutic target.

Less is known about the role of RORγ in NAFLD/NASH. Several studies have highlighted the detrimental role of Th17/IL17 in NASH progression via modulation of hepatic inflammation in mice [135,136]. As RORγ is a key factor involved in Th17 cell differentiation, targeting RORγ with an inverse agonist may have benefits; however, these studies have yet to be completed. NOD-like receptor protein 3 (NLRP3) inflammasome activation occurs in NAFLD, and blocking its activity improves the pathology and fibrosis in MCD-fed mice [137,138,139]. As RORγ inverse agonist SR1001 has been shown to decrease NLRP3-inflammasome activity [140], we can hypothesize that SR1001 may improve NAFLD. The role of RORs in the development of NAFLD/NASH diseases seems to be dependent on the cell type and the stage of the disease. Based on published data, ROR modulators are promising compounds that can be optimized and assessed for their clinical beneficial effects on NAFLD in patients.

### 2.5. REV-ERBs

**Figure 9:**
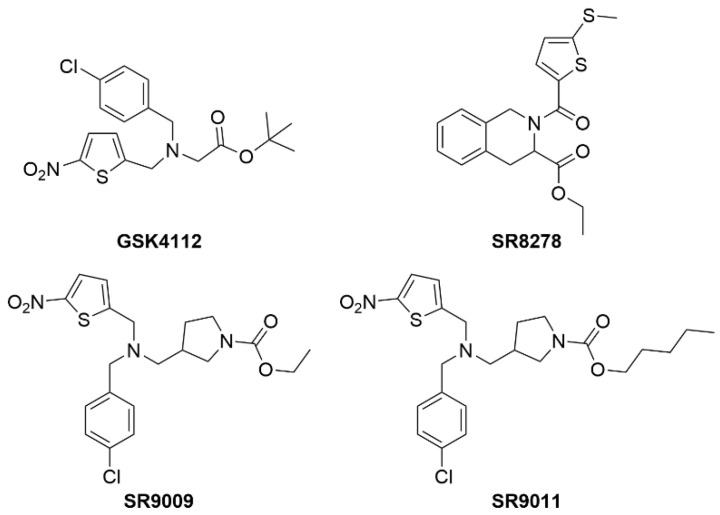
Structures of REV-ERB chemical tool compounds.

*Rev-erbα* (NR1D1) was originally identified based on its canonical NR domain structure. REV-ERBβ (NR1D2) was identified based on its homology to other NRs and has an overlapping pattern of expression with *Rev-erbα*. *Rev-erbα* has been shown to regulate lipid and glucose metabolism via direct regulation of *PEPCK*, *G6pase*, *ApoA1*, *Srebf1*, etc. [141,142,143,144]. In fact, *Rev-erbα*^−/−^ mice display a dyslipidemic phenotype with elevated very-low-density lipoprotein (VLDL) and triglycerides (TG). The Lazar and Evans’ groups have demonstrated the metabolic effects of knocking out *Rev*-*Erb* in mouse models [143,145,146]. REV-ERBs were identified as orphan NRs in the early 1990s, but only recently the natural ligand, heme, was identified by the Burris group [147,148]. Both *Rev-erbα* and –β were originally thought to be constitutive transcriptional repressors; however, the Burris group was the first to demonstrate that this constitutive repression was dependent on the presence of heme [147]. Without heme bound to the LBD, REV-ERBs are transcriptionally inactive. Heme binds to the LBD reversibly with a K_d_ in the range of 1–2μM, which places it in the range of intracellular levels. With the discovery that REV-ERBs can be regulated by a ligand, several groups proceeded to design and characterize synthetic molecules that have potential for modulating glucose and lipid metabolism, and potentially are useful for the study of metabolic diseases. The first synthetic agonist (GSK4112) was described in 2008 and demonstrated modulation of circadian function in tissue culture; however, it has no in vivo activity [149]. Burris and collaborators reported the first synthetic antagonist (SR8278) [150] and agonists (SR9009 and SR9011) [151] that can be used as in vivo tool compounds for the study of REV-ERB-regulated physiology (Figure 9).

**Figure 10:**
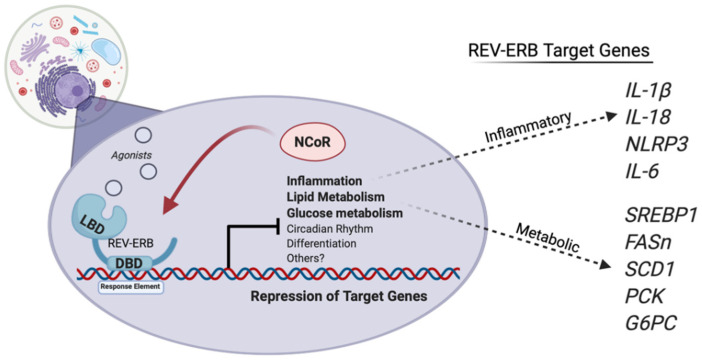
REV-ERBs regulate the transcription of genes involved in the activation of the NLRP3 inflammasome and genes that regulate liver metabolism. REV-ERB activation suppresses proinflammatory genes involved in the progression from steatosis to steatohepatitis, reducing circulating cytokines and inhibiting the activation of the NLRP3 inflammasome. Additionally, activating REV-ERB pharmacologically suppresses lipid and glucose metabolism, and may provide a beneficial effect for those also suffering from hyperglycemia and obesity. Figure created with BioRender (accessed on 7 February 2022).

The REV-ERB receptors are circadian proteins that regulate the expression of the molecular clock. The molecular clock is an anticipatory system that optimizes metabolic processes and behavior to predict environmental changes related to nutritional availability. In mammals, the molecular clock is driven by a transcriptional–translational feedback system that robustly oscillates and repeats itself every 24 h. The transcriptional activators *CLOCK* and *BMAL1* form a heterodimer that binds to E-box motifs within the *PER* and *CRY* genes. The induction of *PER*/*CRY* transcription results in a feedback loop that represses *BMAL1*/*CLOCK* activity. The REV-ERBs and ROR provide further regulation by competitively binding to RORE motifs within the promoter of *BMAL1* and *CLOCK*, further adding intricate feedforward/feedback loop to the molecular clock. Hepatic metabolism and inflammation are innately connected to the molecular clock and, by extension, controlled by the REV-ERBs (Figure 10) [34].

*Rev-erbα* KO mice display perturbed metabolic gene expression with elevated serum levels of increased serum VLDL and triglycerides along with *apoC-III* [152,153]. The ELOVL3 protein is a very-long-chain fatty acid elongase that regulates hepatic lipid metabolism and displays temporal rhythm that is coordinated by *SREBP1* and *Rev-erbα*. Proteolytic activation of *SREBP1* is circadian and integrates diurnal lipogenic and cholesterogenic gene transcription to sense changes in the nutritional state [154]. *Rev-erbα* also indirectly regulates the cyclic activity of *SREBP1* by the cyclic expression of *Insig2* gene, an *SREBP1* sequester protein that is in the endoplasmic reticulum. This in turn interferes with the proteolytic activation of *SREBP1* in the Golgi [155]. *Rev-erbα* KO mice express impaired secretion in the bile and feces with also a reduced bile acid synthesis rate. The expression of the rate-limiting enzyme, cholesterol-7a-hydroxylase (*CYP7A1*), is lowered in *Rev-erbα* KO mice, whereas hepatic overexpression of *Rev-erbα* by adenovirus rescued the expression of *CYP7A1* [142,155,156,157,158]. The specific deletion of *Rev-erbα* and *Rev-erbβ* in the liver perturbs metabolic and cholesterol gene expression and promoted hepatic steatosis [146], demonstrating that *Rev-erbα* regulates the synthesis of cholesterol to bile acids and hepatic lipid metabolism (Figure 10).

The recent development of synthetic REV-ERB agonist SR9009 has provided beneficial evidence of targeting the REV-ERBs to treat metabolic disease and obesity and was the first pan REV-ERB agonist that could be used in vivo. SR9009 treatment in mice lowers triglyceride levels in diet-induced obese mice by decreased lipogenesis and enhanced lipid oxidation [151]. The REV-ERB agonist SR9009 also lowered plasma cholesterol levels in wild-type C57BL/6 and LDLR KO mice and reduced a myriad of cholesterol and bile acid synthesis genes [151,159,160]. Due to REV-ERB being directly involved in the physiological processes in the development of NAFLD, REV-ERB activation in mice placed on a high-fat and high-fructose diet suppressed the progression of fatty liver disease. SR9009 repressed lipogenesis and inflammatory gene expression and, as a result, decreased the development of the disease (Figure 10) [161]. Overall, the data not only suggest that targeting REV-ERB is an effective strategy to lower LDL cholesterol levels, but it also can be utilized to combat the progression of fatty liver disease in an obese state.

Accumulating evidence supports that targeting REV-ERB is a promising approach for management of inflammatory diseases. *Rev-erbα* activation with a synthetic agonist is shown to ameliorate ulcerative colitis, fulminant hepatitis, neuroinflammation, heart failure, myocardial infarction, experimental autoimmune encephalomyelitis, and pulmonary inflammation. Low-grade hepatic inflammation is exhibited by high-fat diet and innate immune cell infiltration. The nuclear glucocorticoid receptor (GR) is a major regulator of metabolism and inflammatory response. Its ligand cortisol has been extensively studied and synthetic derivatives have become one of the most successful classes of anti-inflammatory drugs. GR exhibits a diurnal expression in the liver and becomes more sensitive to cortisol during the light cycle. *Rev-erbα* and GR both physically interact with liver-specific hepatocyte nuclear transcription factors to regulate GR recruitment to the chromatin. The genetic ablation of *Rev-erbα* inverted diurnal GR sensitivity to dexamethasone (dex) and protected the liver from dex-induced lipid accumulation. This suggests that *Rev-erbα* is a direct link to inflammation-driven metabolic dysregulation in the liver [162].

Recently, *Rev-erbα* has been shown to be a regulator of the multimeric protein complex, the NLRP3 inflammasome. The NLRP3 complex induces the release of the proinflammatory cytokines IL-1β and IL-18 and the dysfunction of NLRP3 inflammasome has been implicated in a plethora of diseases [163]. Loss of *Rev-erbα* from primary macrophages displayed altered expression patterns of NLRP3 and IL-1B and 1L-18 production levels. *Rev-erbα* KO mice developed severe acute peritoneal inflammation and fulminant hepatitis induced by a lipopolysaccharide (LPS) endotoxin. Mice treated with SR9009 developed less-severe liver failure and had increased survival times when compared to their controls [164]. The ablation of *Rev-erbα* induced the activation of the NLRP3 inflammasome in mice. *Rev-erbα* inactivated the NLRP3 by suppressing the transcription of p65 and indirectly through the NF-κB pathway. Pharmacological activation of REV-ERB with SR9009 attenuates dextran sulfate sodium-induced colitis and the protected effects were lost by *Nlrp3* and *Rev-erbα* deletion in the mice [165]. Expanding further into cardiac tissue, activation of REV-ERB by only one day abates the activated NLRP3 inflammasome in the cardio-fibroblasts when mice are subjected to ischemia-reperfusion, suggesting long-term benefits in cardiac repair [166].

REV-ERB has demonstrated utility in treating neuroinflammation by the NF-κB pathway. LPS-induced microglial activation-induced damage through NF-κB was attenuated by SR9009 treatment by lowered IL-6 and TNFα secretion [167]. Transcriptomic analysis from *Rev-erbα* KO hippocampus displayed an inflammatory expression signature. *Rev-erbα* KO primary microglia exhibited enhanced NF-κB basal activity and exacerbated oxidative damage in the hippocampal region by peripheral LPS injections. Activation of REV-ERB*α* by SR9009 protected LPS-induced neuroinflammation of the neurons [168]. Providing evidence of the expanding therapeutic potential of REV-ERB agonists in treating a collection of inflammatory linked diseases from hepatic failure, colitis to neuroinflammation. Based on the roles that REV-ERBs play in regulating both metabolism and inflammation, it is possible that targeting REV-ERBs to suppress lipogenesis and/or inflammatory pathways, that include NLRP3 and NF-κB, with selective agonists may be beneficial and provide therapeutic utility for the treatment of NAFLD.

### 2.6. ERRs

The estrogen-receptor-related orphan receptors (ERRα, ERRβ, and ERRγ) were the first orphan nuclear receptors to be identified [169]. As their name indicates, they are homologous to estrogen receptors (ERα and ERβ); however, their ligand-binding domain does not bind endogenous ER ligands. While ERs require ligand binding to display transcriptional activity, all three ERRs exhibit ligand-independent constitutive transcriptional activity [170]. Unlike ERs that function as obligate homodimers, ERRs function as monomers and bind to a DNA response element that is distinct from the classical palindromic ER DNA response elements [170]. ERRs are highly expressed in tissues with high energy demand such as the skeletal muscle, heart, brain, adipose tissue, and liver [169,171,172]. A range of target genes have been identified that includes enzymes and regulatory proteins in energy production pathways involved in fatty acid oxidation, the tricarboxylic acid (TCA) cycle, mitochondrial biogenesis, and oxidative phosphorylation (OXPHOS) [173,174].

**Figure 11:**
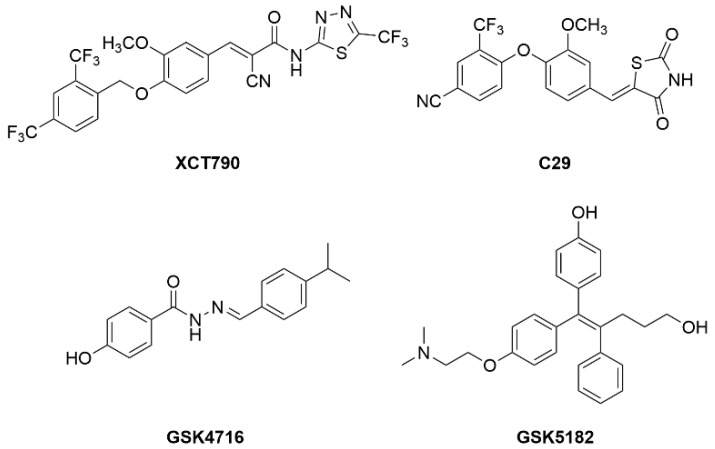
Chemical structures of known ERR modulators.

Several cofactors have been identified for ERRs, such as nuclear respiratory factor 1 (NRF-1), members of the MEF2 family (myocyte enhancer factor 2) [175,176], or peroxisome proliferator-activated receptor g coactivator-1 alpha or beta (PGC-1α or PGC-1β). ERRs are constitutively active and no endogenous ligands have been characterized yet. Crystal structures of ERRα LBD and other pharmacological studies have described several synthetic ligands of ERRs. Since the first ERRα inverse agonist XCT790 was discovered [177], other studies have described ERR synthetic ligands and their roles in vivo. For example, ERRα inverse agonist (C29) displays an anti-diabetic activity in rodent models [178,179], GSK4716 (ERRβ/γ agonist) increases mitochondrial function in mouse myotubes in vitro [180]. GSK5182, an ERRγ inverse agonist, was demonstrated to have anti-hyperglycemic effect in obese mice action via suppression of gluconeogenesis (Figure 11) [181].

Embryonic lethality in ERRβ-null mice [182] and death of ERRγ-null mice before 1 week of age [176] has severely limited our understanding of the role of these receptors in metabolic regulation. In the liver, ERRα, ERRγ, and PGC1α are upregulated upon fasting and have been shown to bind several target genes involved in mitochondria oxidation and gluconeogenesis [181]. Several studies described the role of ERRs in regulation of mitochondria biogenesis, electron transport chain (ETC), OXPHOS, fatty acid b-oxidation (FAO), or glucose metabolism in liver [183,184]. Liver ChIP-seq data has shown ERRα as a key regulator of TCA cycle and lipid metabolism [185]. ERRα induces the expression of phosphoenolpyruvate carboxykinase 1 (*PCK1*) and glucose-6-phosphatase (*G6PC*) gene expression, whereas ERRγ inhibits their expression, providing a mechanism by which the isoforms have opposing effects on gluconeogenesis [176,181,182,183,184,185,186]. ERRγ overexpression in the liver induces gluconeogenic genes and increases serum glucose in fasted mice [107]. Surprisingly, ERRα contributes to the development of NAFLD in a context-dependent manner. ERRα^−/−^ mice display an unexpected phenotype of reduced body and fat mass and resistance to weight gain on a high-fat diet (HFD) and decreased intrahepatic lipid accumulation [187]. While the expression of ERRα- and PGC-1α-encoding genes are upregulated in WT mice under HFD, this response is likely an adaptive response to mitochondrial dysfunction [188]. Genetic (ERRα^−/−^) or pharmacological inhibition (synthetic inverse agonist C29) exacerbates rapamycin-induced NAFLD in mice [185] and impairs the reversal of fasting-induced NAFLD during refeeding [189]; activating ERRα appears more beneficial to treat and reverse the instilled disease.

Altogether, these data clearly define ERR as a potential target for NAFLD. Very few ERR agonists have been described, and no data is currently available to really address the efficiency of the ERRs to protect or prevent NAFLD.

## 3. Conclusions and Perspectives

Metabolic syndrome exhibits a collection of diseases that include high blood pressure, insulin resistance, obesity, and elevated LDL cholesterol and triglycerides. Although difficult to diagnose, NAFLD has been found to be an additional comorbidity that exacerbates the metabolic syndrome and, if left unchecked, will produce devastation to the patient’s health. Remarkable progress has been made in the discovery and improvement of synthetic ligands for the metabolic NRs ERRs, REV-ERBs, RORs, FXR, PPARs, and LXR, and all have supplied undeniable therapeutic evidence in obesity models. It is worth noting that this review only focused on a few of the NRs that play a role in the metabolic and/or inflammatory regulation of liver disease. Other receptors, such as the pregnane X receptor (PXR), estrogen receptor (ER), constitutive androstane receptor (CAR), and others, may also hold therapeutic potential in these diseases. Further evaluation of the NRs and their ligands in NASH models will provide further insights for drug discovery and physiological relevance of these receptors in fatty liver diseases.

In the past years, the reported NR ligands have been tested in animal models of metabolic disease, and recently some NR ligands have been pursued with clinical success. The advancement towards improved diagnostic technology and an understanding the etiology of NAFLD has provided new strategies to treat the disease. With the advancement with metabolic NR ligands and a stronger understanding of their regulation of liver physiology, exciting new strategies to treat NAFLD are presented. The exponential increase in NAFLD cases worldwide clearly indicates that new strategies are needed to blunt the trajectory. Only by exhaustively exploring all therapeutic options, including NR pharmacology, will NAFLD be controlled with a sequential profound improvement to worldwide health.

## Figures and Tables

**Table 1:** Summary of NR compounds in NAFLD and NASH studies. Up arrow (↑) indicates an increase while a down arrow (↓) indicates a decrease.

FXR	Cilofexor (phase 2)	FXR agonist	↓ Serum bile acids ↓ Hepatic Steatosis
	Cilofexor + Firsocostat (phase 2)	FXR agonist + *ACC* inhibitor	↓ Hepatic Steatosis ↓ Liver Stiffness ↓ ALT
	Cilofexor + Firsocostat + Selonsertib (phase 2)	FXR agonist + *ACC* inhibitor + ASK1 inhibitor	Currently ongoing and awaiting results
	TERN-101/LY2562175 (phase 2)	FXR agonist	↓ LDL ↓ TGs ↑ HDL
	EDP-305 (phase 2a)	FXR agonist	↓ Hepatic steatosis ↓ ALT Side effects including pruritus, headaches, and GI issues were reported
	GW4064	FXR agonist	↓ Hepatic steatosis ↓ Hyperglycemia Poor bioavailability
	Obeticholic Acid (phase 2)	FXR agonist	↓ Hepatic inflammation ↓ Fibrosis Observed increases in LDL in some patients but can be co-treated with statins
PPAR	Thiazolidinediones (TZDs), (FDA approved for diabetes; phase 2 for NASH)	PPARγ agonist	↑ Insulin sensitivity ↑ Peripheral glucose clearance ↓ Hepatic steatosis ↓ FFA
	Seladelpar (phase 2)	PPARδ agonist	↓ ALT ↓ LDL Terminated due to increased liver damage
	Saroglitazar (phase 2 for NAFLD; phase 3 for NASH)	PPARα/δ agonist	↓ Hepatic steatosis ↓ Liver enzymes Approved in India for use in NASH
	Lanifibranor (phase 3)	Pan-PPAR agonist	↓ Hepatic inflammation
	GW501516	PPARδ agonist	↑ Insulin sensitivity ↓ Hepatic steatosis Induced cancer in preclinical models
LXR	T0901317	LXRα/β agonist	↓ Cellular cholesterol ↑ Cholesterol Efflux ↑ Hepatic lipogenesis Initially in clinical trials for atherosclerosis but removed due to increased hepatic steatosis
	GW3965	LXRα/β agonist	↑ Glucokinase expression ↓ Gluconeogenesis ↓ Inflammation ↑ Plasma and liver TGs
	LXR-632 (phase 1)	LXRα/β agonist	↑ Anti-atherogenic properties Terminated post-phase 1 due to treatment-emergent adverse events
	CS-8080 (phase 1)	LXRα/β agonist	Clinical trials were terminated due to undisclosed reasons for these compounds.
	BMS-779788 (phase 1)	LXRα/β agonist	
	BMS-852927 (phase 1)	LXRα/β agonist	
	AHRO-001 (phase 1)	LXRα/β agonist	↑ HDL ↑ Anti-atherogenic properties
	SR9238	Liver-specific LXRα/β inverse agonist	↓ Hepatic steatosis ↓ Hepatic inflammation
	SR9243	LXRα/β inverse agonist	↓ Hepatic steatosis ↓ Hepatic inflammation Targets Warburg effect in cancer cells
ROR	SR1078	RORα/γ agonist	↑ *FGF21* expression ↑ *G6Pase* expression
	SR1001	RORα/γ inverse agonist	↓ Th17 cell-driven hepatic inflammation
REV-ERB	GSK4112	*Rev-erbα*/β agonist	No in vivo activity
	SR8278	*Rev-erbα*/β antagonist	Not tested in NAFLD but drives muscle regeneration and improves glucose regulation via increased osteocyte turnover
	SR9009	*Rev-erbα*/β agonist	↓ Plasma cholesterol ↓ Hepatic fibrosis ↑ Lean muscle mass ↓ Fat mass ↓ Activation and expression of NLRP3 inflammasome
ERR	XCT790	ERRα inverse agonist	Anti-diabetic activity in rodents
	GSK4716	ERRβ/γ agonist	↑ Mitochondrial function in myotubes
	GSK5182	ERRγ inverse agonist	↓ Plasma glucose in obese mice

## Abbreviations

NAFLD, Non-Alcoholic Fatty Liver Disease; NASH, Non-Alcoholic Steatohepatitis; HCC, Hepatocellular Carcinoma; BMI, Body Mass Index; ROS, Reactive Oxygen Species; IL-1b, Interleukin 1 beta; IL-18, Interleukin 18; NFkB, Nuclear factor kappa-light-chain-enhancer; MAP, mitogen-activated protein; NLR, NOD-like receptor; NLRP1, NOD-like receptor protein 1; NLRP3, NOD-like receptor protein 3; NLRC4, NLR-family CARD-domain containing 4; PAMP, Pathogen-associated molecular patter; DAMP, Damage-associated molecular pattern; HSCs, Hepatic Stellate Cells; NRs, Nuclear Receptors; DBD, DNA-binding domain; LBD, Ligand-binding domain; FXR, Farnesoid X Receptor; RXR, Retinoid X Receptor; IR1, Inverted repeats with 1 nucleotide separation; CDCA, chenodeoxycholic acid; CA, cholic acid; VLDL, Very Low-Density Lipoprotein; FGF19, Fibroblast Growth Factor 19; *CYP7A1*, Cytochrome P450 Family 7 Subfamily A Member 1; *SREBP1c*, sterol regulatory element binding protein-1; SHP, Small heterodimer partner; PGC1a, Peroxisome proliferator-activated receptor gamma coactivator 1-alpha; *PEPCK*, Phosphoenolpyruvate carboxykinase; *G6Pase*, Glucose 6-phosphatase; CREB, cAMP response element-binding protein; GR, Glucocortecoid Receptor; HNF4, Hepatic Nuclear Factor 4; MCP-1, monocyte chemoattractant protein-1; LDL, Low-density lipoprotein; *ACC*, acetyl-CoA carboxylase; PDFF, proton density fat fraction; ASK1, apoptosis signal-regulating kinase-1; PBC, Primary biliary cholangitis; HDL, High density lipoproteins; GI, Gastrointestinal; PPAR, Peroxisome proliferator-activated receptors; DR1, direct repeat 1; TZD, thiazolidinediones; T2DM, Type 2 diabetes mellitus; FFA, Free fatty acid; PSC, Primary Sclerosing Cholangitis; ALT, alanine aminotransferase; LXR, Liver X Receptor; LXRE, LXR Response Element; Gal4, galactose-responsive transcription factor; DR4, direct repeat 4; RAR, Retinoid Acid Receptor; *ABCA1*, ATP Binding Cassette Subfamily A Member 1; *FASn*, fatty acid synthase; *ChREBP*, carbohydrate response-element binding protein; *SCD1*, Stearoyl–CoA desaturase; *ARL7*, ADP-ribosylation factor-like 7; ROR, retinoid acid receptor-related receptor; PXR, Pregnane X receptor; APOD, Apolipoprotein D; SPOT14, Thyroid Hormone-Inducible Hepatic Protein; *GLUT4*, Glucose transporter type 4; AE, Adverse Events; TEAE, Treatment emergent adverse events; RORE, ROR Response Element; *FGF21*, Fibroblast Growth Factor 21; Th17, T-helper 17; CD4, cluster of differentiation 4; CD8, cluster of differentiation 8; M2, M2-type macrophage (anti-inflammatory); IL-17, Interleukin 17; MCD, Methionine-Choline Deficient; *ApoA1*, Apolipoprotein A1; KO, knock out; TG, Triglycerides; ERR, estrogen receptor-related receptor; NRF-1, nuclear respiratory factor 1; MEF2, myocyte enhancer factor 2; ETC, electron transport chain; TCA, tricarboxylic acid cycle; OXPHOS, oxidative phosphorylation; FAO, fatty acid oxidation; *PCK1*, phosphoenolpyruvate carboxylase 1; *G6PC*, glucose-6-phosphatase.
